# Knowledge structure and future research trends of body–mind exercise for mild cognitive impairment: a bibliometric analysis

**DOI:** 10.3389/fneur.2024.1351741

**Published:** 2024-01-23

**Authors:** Jing Zhang, Zhen Yang, Huiying Fan

**Affiliations:** ^1^Faculty of Physical Education, Shanghai International Studies University, Shanghai, China; ^2^Faculty of Movement and Rehabilitation Sciences, KU Leuven, Leuven, Belgium; ^3^Lothian Birth Cohort, Department of Psychology, The University of Edinburgh, Edinburgh, United Kingdom; ^4^School of Physical Education, Shanghai Normal University, Shanghai, China

**Keywords:** body–mind exercise, mild cognitive impairment, knowledge structure, research trends, bibliometric analysis

## Abstract

**Background:**

Mild cognitive impairment (MCI) is a common neurodegenerative disorder that poses a risk of progression to dementia. There is growing research interest in body–mind exercise (BME) for patients with MCI. While we have observed a rapid growth in interest in BME for MCI over the past 10 years, no bibliometric analysis has investigated the knowledge structure and research trends in this field. Consequently, the objective of this research is to conduct a bibliometric analysis of global publications of BME for MCI from 2013 to 2022.

**Methods:**

A total of 242 publications in the field of BME for MCI were retrieved from the Web of Science Core Collection. Bibliometric analysis, including performance analysis, science mapping, and visualization, was performed using CiteSpace, VOSviewer, and Microsoft Excel.

**Results:**

Publications and citations in the field of BME for MCI have shown a rapidly increasing trend over the last decade. Geriatrics & Gerontology, and Neurosciences were the most frequently involved research categories. China (78 documents) and the USA (75 documents) contributed to the largest number of publications and had the strongest international collaborative networks. Fujian University of Traditional Chinese Medicine contributed to the largest number of publications (12 documents), and Chen, L of this institution was the most prolific author (12 documents). Frontiers in Aging Neuroscience (16 documents), and JOURNAL OF ALZHEIMER’S DISEASE (12 documents) were the most prolific journals. Tai Chi and Baduanjin, as specific types of BME, were the hotspots of research in this field, while evidence synthesis and guidelines might be future research trends.

**Conclusion:**

In the last decade, there has been a rapid growth in scientific activities in the field of BME for MCI. The results of this study provide researchers and other stakeholders with knowledge structure, hotspots, and future research trends in this field.

## 1 Introduction

As global aging accelerates, cognitive decline, including mild cognitive impairment (MCI), has attracted growing concerns from researchers, clinical practitioners, as well as a wide range of other stakeholders (1). As a status between normal cognitive aging and dementia, MCI has emerged as a priority in both research and clinical practice to delay the development of neurodegenerative disease (2). MCI typically consists of memory impairments accompanied by abnormal memory test scores, affecting professional and social activities, but with maximum preservation of activities of daily living (3). The latest meta-analysis proposed that 15.6% of adults over 50 years old were suffering from MCI (4), while the incidence of MCI was 22.5, 40.9, and 60.1% in people aged 75–79 years, 80–84 years, and over 85 years, respectively (5). Due to the natural trajectory of cognitive decline, aging is a risk factor for MCI, while males are more prone to suffer from MCI than females (6). More importantly, MCI carries a varying cognitive developmental trajectory, including a reversal to normal cognitive function, retention of stability, and progression to dementia (7). Previous research suggested that approximately 80% of patients with MCI may have converted to Alzheimer’s disease with annual conversion rates around 10–15% (8–10). Meanwhile, it was reported that 18% of patients reversed from MCI to normal cognitive function (11). Nevertheless, the risk of cognitive decline is still higher for patients with MCI than for people with a normal trajectory of cognitive decline (12). Furthermore, patients with MCI are commonly accompanied by a variety of neuropsychiatric symptoms and reduced ability to perform activities of daily living (13). The average annual direct medical costs for an individual with MCI in the USA were estimated at $6,499, which is substantially higher than those without MCI ($2,969) (14). Therefore, MCI, as a prevalent cognitive disorder among middle-aged and elderly people, needs to be prevented and treated with more action, especially in this aging world.

Even though a number of pharmacological interventions have been developed to treat MCI (15–17), none of them has been reported to be effective (7). Meanwhile, exercise has shown potential value as a non-invasive and highly feasible non-pharmacological treatment for patients with MCI (18). The body–mind exercise (BME), which combines body movement, mental focus, and controlled breathing to improve strength, balance, flexibility, and overall health (19), has been reported to benefit on cognitive disorders such as MCI among middle-aged and elderly adults (20–22). Fabre et al. (23) proposed that combined aerobic and mental training was more effective than separate aerobic or mental training in memory quotient among healthy older adults. Similarly, Theill et al. (24) found that 10 weeks of combined cognitive and physical training was more effective for cognitive performance than single cognitive training among older adults. More recently, Tai Chi, Yoga, Qigong, and other types of BME have been increasingly employed to enhance cognitive function and to manage MCI among older adults (25–27).

As mentioned above, researchers have conducted quite a few in-depth original investigations of BME for MCI, and this has resulted in several evidence syntheses, such as systematic reviews, and meta-analyses (28, 29). These kinds of syntheses employ systematic approaches that allow for the robust extraction of qualitative or quantitative information from publications and then identify the existing evidence on various specific research questions (30). However, these syntheses are not suitable for a broad and rapidly developing research field and fail to deal with highly heterogeneous publications (30). For instance, in the field of BME for MCI, the original investigations include randomized controlled trials and laboratory mechanistic studies. Existing approaches, whether systematic reviews or meta-analyses, are unable to synthesize evidence from interventions’ effectiveness and evidence from imaging reports simultaneously.

Therefore, Nakagawa et al. (31) proposed a “Research Weaving” framework that combines bibliometrics and systematic mapping to reveal and visualize the knowledge structure and research trends in a research field. The major advantage of this approach is that it allows for the synthesis of a large number of heterogeneous scientific publications, thus providing better insights into the knowledge structure and future research trends (30). Considering these advantages, bibliometric analyses have begun to become a standard instrument in science policy and research management (32). In research fields related to BME for MCI, such as exercise for Parkinson’s Disease (33), and neuroinflammation-induced MCI (34), bibliometric analysis has provided a one-stop overview of the field, as well as the identification of knowledge gaps. Nevertheless, there is an absence of bibliometric analysis in the field of BME for MCI. Therefore, a robust bibliometric analysis of BME for MCI, which is a rapidly growing field with highly heterogeneous publications, is required to support researchers to identify future research directions and to provide quantitative evidence for policy makers to identify future funding priorities.

Accordingly, this research aims to assess the knowledge structure of the field of BME for MCI from 2013 to 2022, and to predict future research directions. Specifically, this present research will answer the following research questions: (1) Which country, institution, author, discipline, journal, and reference significantly contributed to the research in the field of BME for MCI? (2) What are the research trends in the field of BME for MCI from 2013 to 2022? (3) What are the hotspots of research in the field of BME for MCI?

## 2 Methods

### 2.1 Data source and search strategy

This study was conducted in rigorous accordance with the step-by-step guidelines for bibliometric analysis (30).

All eight indexes of the Web of Science Core Collection (WoSCC) were utilized as an electronic database for this bibliometric analysis. The rationale for WoSCC is that, as this study aims to perform a bibliometric analysis of the multidisciplinary field of BME for MCI, the usage of specialist field databases such as PubMed and SPORTDiscus may result in an omission of search results. Furthermore, using multiple databases can introduce bias into subsequent bibliometric analyses because of the differences in data formats and the included information of various databases (e.g., the difference between WoSCC and Scopus^@^ regulations on author initials) (35). As WoSCC provides the most extensive information for bibliometric analysis compared to other multidisciplinary electronic databases such as Google Scholar and Scopus@, WoSCC was chosen as the only database in this study (36). Moreover, WoSCC has been successfully used by researchers as a data source to conduct bibliometric analysis in the field of Tai Chi for Health (37), electroencephalogram research in MCI (38), and other fields related to BME for MCI.

**Figure 1:**
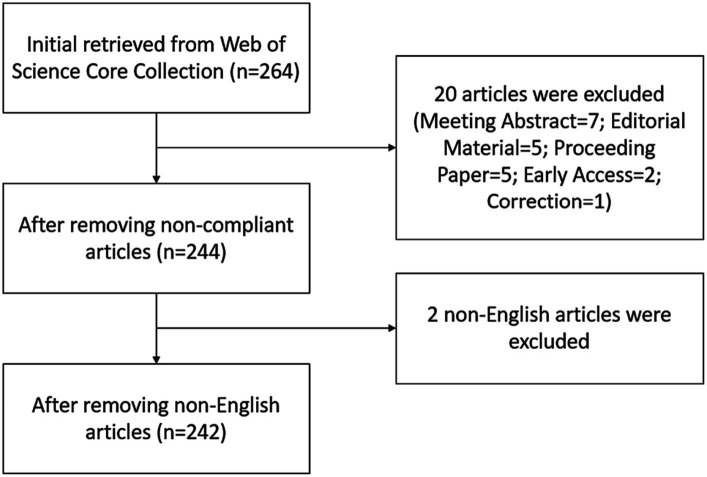
Flow chart of the literature search in the field of body–mind exercise for patients with mild cognitive impairment.

As recommended by the guidelines for conducting bibliometric analyses, the search strategy used for this study was defined by the keywords used in the previous reviews in the field of BME for MCI, as well as brainstorming by all the authors (30). Taking into account the update of the WoSCC electronic database, all data were retrieved on March 13, 2023, to eliminate search bias. Keywords related to BME were entered in the topic column and recorded as #1, while keywords related to MCI were entered in the topic column and recorded as #2. The final search strategy is available in Supplementary material. The document type was set as “Article” and “Review Article,” the timespan was set as 2013–2022, and the language of publication was limited to English. The reason for limiting the search to the last 10 years is that the BME for MCI research field has developed over a relatively short period and that 10 years of bibliometric analyses have already demonstrated their ability to detect the knowledge structure and to predict research trends in previous studies (39). The flow chart of the literature search was shown in Figure 1. This search strategy resulted in 242 eligible documents, and basic information about each document was downloaded from the electronic database in formats of plain text files and tab-delimited files.

### 2.2 Analysis tool

To perform this bibliometric analysis, three software programs, CiteSpace, VOSviewer, and Microsoft Excel, were used to analyze the data downloaded from WoSCC (40, 41). CiteSpace is a Java-based software that has been used extensively in bibliometric analysis and knowledge map visualizations. In this research, the reference co-citation network, and the co-occurring author keyword network in CiteSpace were applied to generate maps of knowledge structures of BME for MCI. As a key function of co-citation networks and co-occurring keyword networks that can characterize the intensity and frequency of mentions of an item over time (42), citation bursts were employed to describe research trends in this study. Due to its advantages in generating visual graphs and handling big data in bibliometric analysis, VOSviewer was used in this study to generate the collaborative networks of countries, institutions, and authors who published in the field of BME for MCI. VOSviewer automatically calculated the total link strength (TLS), which represents the number of co-occurrences of two items (countries/institutions/authors) in publications. Meanwhile, the average citations per item (ACI) was derived by dividing the total number of citations for the item automatically generated by VOSviewer by the number of publications of the item. Microsoft Excel was used to generate publication and citation trends in the field of BME for MCI.

## 3 Results

### 3.1 Annual publication activity

**Figure 2:**
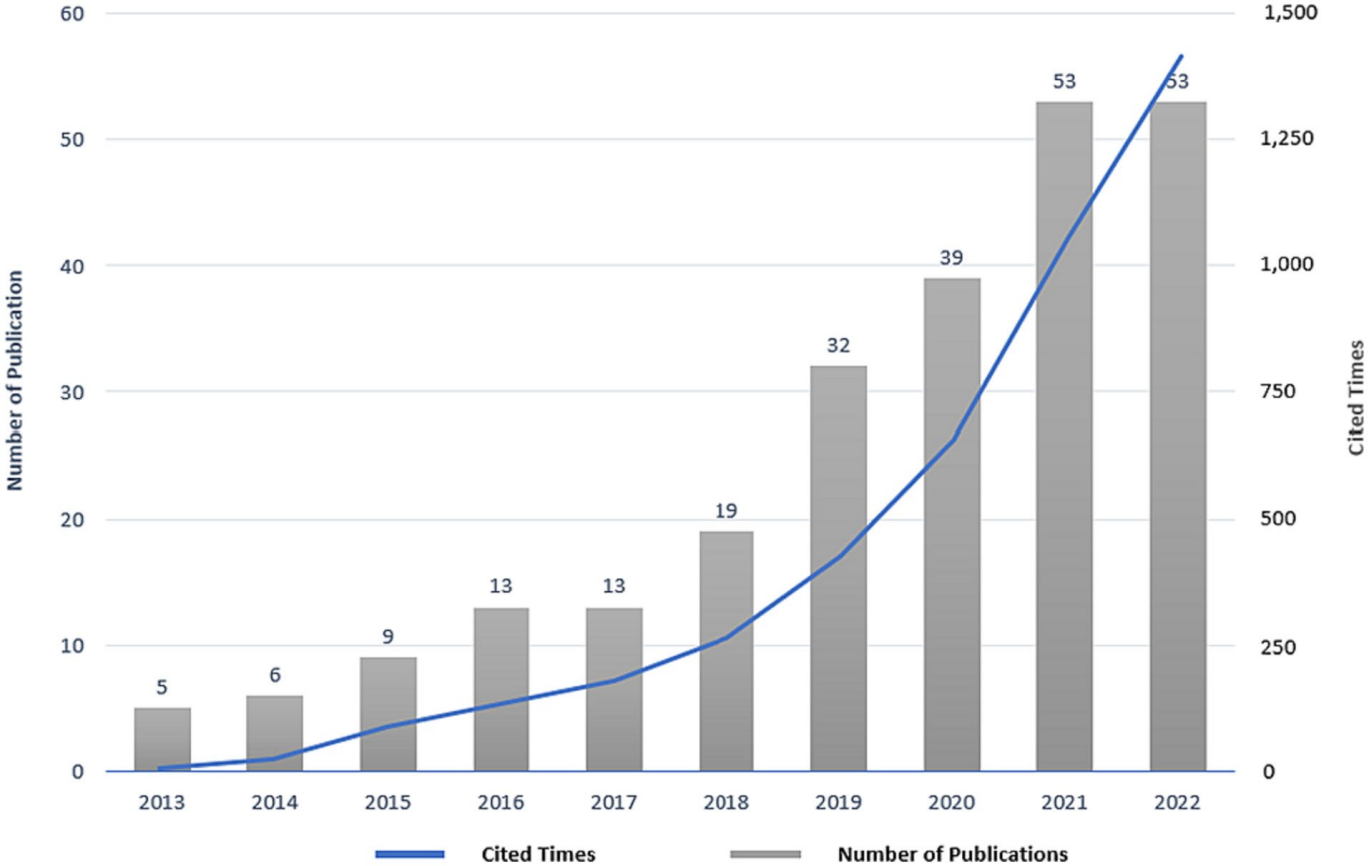
Annual distribution of publications and citations in the field of body–mind exercise for patients with mild cognitive impairment.

From 2013 to 2022, a total of 242 eligible publications were retrieved in the field of BME for MCI, which received a total of 4,118 citations, with 17.02 citations per publication. Among the 242 publications in the field of BME for MCI, 143 publications were “Article,” and 99 publications were “Review Article.” Figure 2 clearly illustrates the rapid upward trend in the past 10 years in both publications and citations in the field of BME for MCI. Annual publications in this field topped 10 for the first time in 2016 and then peaked in 2021 and 2022 (53 publications). Meanwhile, annual citations also grew rapidly after first exceeding 100 times in 2016 and then surpassing 1,000 times in 2021 until peaking in 2022 (1,382 times). In addition, 182 of the 242 publications in this field over the past decade received at least one grant (75.21%) and were published in 50 Web of Science categories. Geriatrics & Gerontology (72 publications, 29.75%), Neurosciences (49 publications, 20.25%), Psychiatry (33 publications, 13.64%), Gerontology (31 publications, 12.81%), and Clinical Neurology (22 publications, 9.09%) were the top five categories that had the largest number of publications in the field of BME for MCI.

### 3.2 Analysis of national and institutional productivity

**Figure 3:**
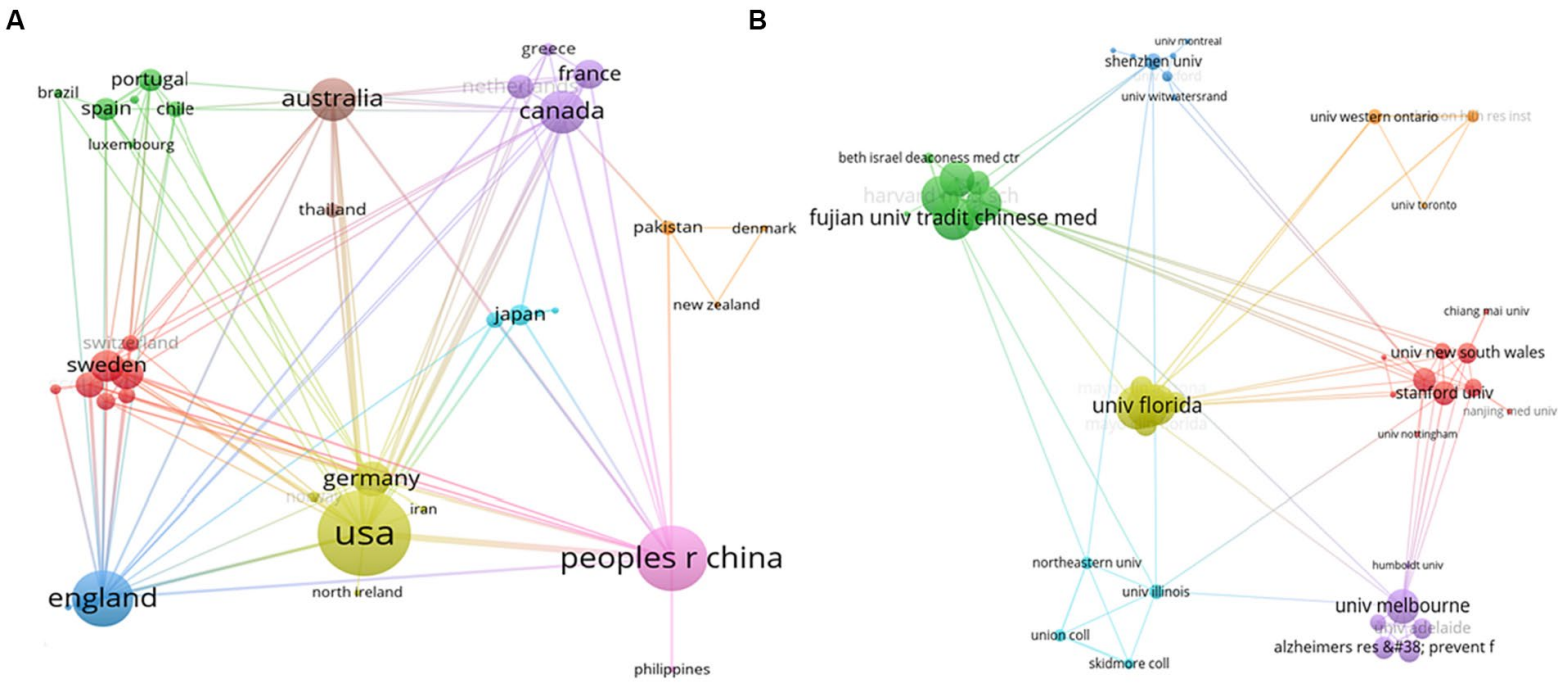
**(A)** Inter-country collaborative network in the field of body–mind exercise for patients with mild cognitive impairment. **(B)** Inter-institutional collaborative network in the field of body–mind exercise for patients with mild cognitive impairment.

According to the results from WoSCC, a total of 42 countries/regions, as well as 635 institutions contributed to at least one publication in the field of BME for MCI in the past decade. Table 1 shows the countries and institutions that contributed to the largest number of publications in the field of BME for MCI from 2013 to 2022. China, with 78 publications (32.23%), and the USA, with 75 publications (30.99%), were the top two countries that published the largest number of documents in the field of BME for MCI and were well ahead of other countries. Furthermore, of the 11 countries with the highest scientific productivity in the field of BME for MCI, only China, Brazil, and Thailand are developing countries. Germany (ACI = 48.10), Thailand (ACI = 36.88), and Australia (ACI = 31.88) had the highest ACI, demonstrating the higher quality of their research in the field of BME for MCI. The USA and China held the strongest collaborative networks, and England, Canada, and Australia also had strong collaborations with other countries. Among the 12 institutions with the highest scientific productivity in the field of BME for MCI, eight are from the USA, three from China, and one from England. The Fujian University of Traditional Chinese Medicine in China contributed to the largest number of publications (12 publications, 4.96%) in the field of BME for MCI, followed by the Harvard Medical School in the USA (10 publications, 4.13%). Massachusetts General Hospital (ACI = 57.71) and the University of California, Los Angeles (ACI = 38.17) in the USA had the highest ACI, proposing that the two institutions had an early start and a high quality of research in the field of BME for MCI. Furthermore, the Fujian University of Traditional Chinese Medicine, the University of Florida, and the University of Washington had the strongest collaborative networks (TLS = 23) in the field of BME for MCI, whereas some institutions had very low collaborative intensity. The inter-country collaborative network in the field of BME for MCI is presented in Figure 3A, while Figure 3B shows the inter-institutional collaborative network in this field.

**Table 1:** Top 11 active countries and top 12 active institutions in the field of body–mind exercise for patients with mild cognitive impairment.

Rank	Country	Quantity	%	ACI	TLS	Rank	Institution	Quantity	%	Country	ACI	TLS
1	China	78	32.23	11.12	44	1	Fujian University of Traditional Chinese Medicine	12	4.96	China	20.33	23
2	USA	75	30.99	23.81	62	2	Harvard Medical School	10	4.13	USA	28.00	21
3	England	19	7.85	15.74	36	=3	University of Florida	8	3.29	USA	27.13	23
4	Canada	18	7.44	28.11	25	=3	The Chinese University of Hong Kong	8	3.29	China	9.50	1
5	Australia	16	6.61	31.88	25	5	Massachusetts General Hospital	7	2.89	USA	57.71	19
6	Italy	13	5.37	20.23	8	=6	University of Washington	6	2.48	USA	24.00	23
=7	Germany	10	4.13	48.10	19	=6	University of Nebraska Medical Center	6	2.48	USA	9.50	21
=7	Japan	10	4.13	10.80	10	=6	Stanford University	6	2.48	USA	27.0	12
9	Spain	9	3.72	21.56	11	=6	University College London	6	2.48	England	9.67	12
=10	Brazil	8	3.31	20.38	3	=6	University of California, Los Angeles	6	2.48	USA	38.17	11
=10	Thailand	8	3.31	36.88	6	=6	Mayo Clinic	6	2.48	USA	9.67	10
						=6	Shanghai University of Sport	6	2.48	China	5.00	0

%, percentage of the quantity of work per country/institution out of the 242; ACI, average citations per item; TLS, total link strength; =, same quantity of publications.

### 3.3 Analysis of authors’ productivity

**Figure 4:**
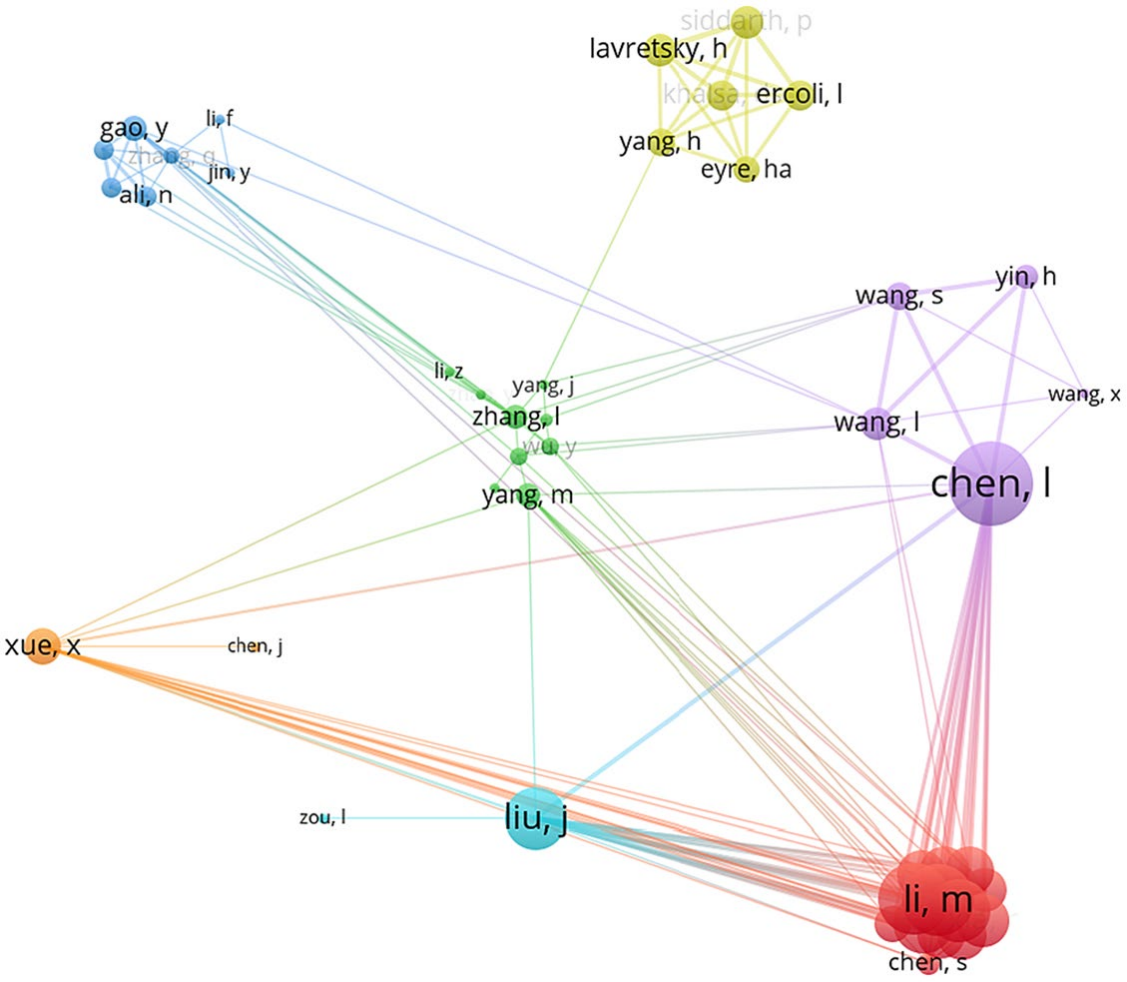
Inter-authorial collaborative network in the field of body–mind exercise for patients with mild cognitive impairment.

A total of 1,357 authors contributed to the 242 publications in the field of BME for MCI in the past decade, and Table 2 shows the 13 active authors with the largest number of publications. Chen, L of Fujian University of Traditional Chinese Medicine was the most prolific author in this field (12 publications, 4.96%), and the only author with more than 10 publications. Khalsa, DS of the Alzheimer’s Research & Prevention Foundation (ACI = 40.20), and Lavresky, H of the University of California, Los Angeles (ACI = 38.17), had the highest ACI in the field of BME for MCI, proposing that their research had high quality. Chen, L (TLS = 68), Li, M (TLS = 55), and Tao, J (TLS = 55) had the strongest collaborative networks. The inter-authorial collaborative network in the field of BME for MCI is illustrated in Figure 4.

**Table 2:** Top 13 active authors in the field of body–mind exercise for patients with mild cognitive impairment.

Rank	Author	Institution	Country	Quantity	%	ACI	TLS
=1	Chen, L	Fujian University of Traditional Chinese Medicine	China	12	4.96	23.75	68
=2	Li, M	Fujian University of Traditional Chinese Medicine	China	7	2.89	31.29	55
=2	Tao, J	Fujian University of Traditional Chinese Medicine	China	7	2.89	30.71	55
=2	Zheng, G	Shanghai Institute of Health Sciences	China	7	2.89	27.14	46
=2	Wang, L	Jilin University	China	7	2.89	12.00	19
=6	Liu, J	Fujian University of Traditional Chinese Medicine	China	6	2.48	30.83	45
=6	Xia, R	Fujian University of Traditional Chinese Medicine	China	6	2.48	19.00	36
=6	Lavresky, H	University of California, Los Angeles	USA	6	2.48	38.17	19
=6	Zhang, L	Jilin University	China	6	2.48	7.00	13
=10	Siddarth, P	University of California, Los Angeles	USA	5	2.07	30.40	19
=10	Khalsa, DS	Alzheimer’s Research & Prevention Foundation	USA	5	2.07	40.20	18
=10	Wang, S	Jilin University	China	5	2.07	18.00	16
=10	Yang, H	University of California, Los Angeles	USA	5	2.07	31.20	16

%, percentage of the quantity of work per author out of the 242; ACI, average citations per item; TLS, total link strength; =, same quantity of publications.

### 3.4 Journal characteristics

A total of 242 publications in the past decade in the field of BME for MCI were published by 127 journals, while the top 14 journals which published the largest number of documents are presented in Table 3. Frontiers in Aging Neuroscience (16 publications, 6.61%), and JOURNAL OF ALZHEIMER’S DISEASE (12 publications, 4.96%) were the two journals publishing the largest number of documents in the field of BME for MCI. Meanwhile, Frontiers in Aging Neuroscience [Impact factor (IF) = 5.702], and Frontiers in Psychiatry (IF = 5.435) were the only two journals with an IF of more than 5, suggesting that publishing research in the field of BME for MCI in high quality journals is still challenging.

**Table 3:** Top 14 active journals in the field of body–mind exercise for patients with mild cognitive impairment.

Rank	Journal	Quantity	%	IF	Country
1	Frontiers in Aging Neuroscience	16	6.61	5.702	Switzerland
2	JOURNAL OF ALZHEIMER’S DISEASE	12	4.96	4.16	Netherlands
3	Frontiers in Psychology	9	3.72	4.232	Switzerland
=4	Geriatric Nursing	7	2.89	2.525	USA
=4	International Journal of Environmental Research and Public Health	7	2.89	4.614	Switzerland
=4	JMIR Serious Games	7	2.89	3.364	Canada
7	MEDICINE	6	2.48	1.817	USA
=8	Frontiers in Human Neuroscience	5	2.07	3.473	Switzerland
=8	Trials	5	2.07	2.728	England
=10	AGING CLINICAL AND EXPERIMENTAL RESEARCH	4	1.65	4.481	Italy
=10	Clinical Interventions in Aging	4	1.65	3.829	New Zealand
=10	Evidence-based Complementary and Alternative Medicine	4	1.65	2.65	England
=10	Frontiers in Psychiatry	4	1.65	5.435	Switzerland
=10	INTERNATIONAL JOURNAL OF GERIATRIC PSYCHIATRY	4	1.65	3.85	England

%, percentage of the quantity of work per journal out of the 242; IF, 2021 Impact Factors.

### 3.5 Keyword analysis

**Figure 5:**
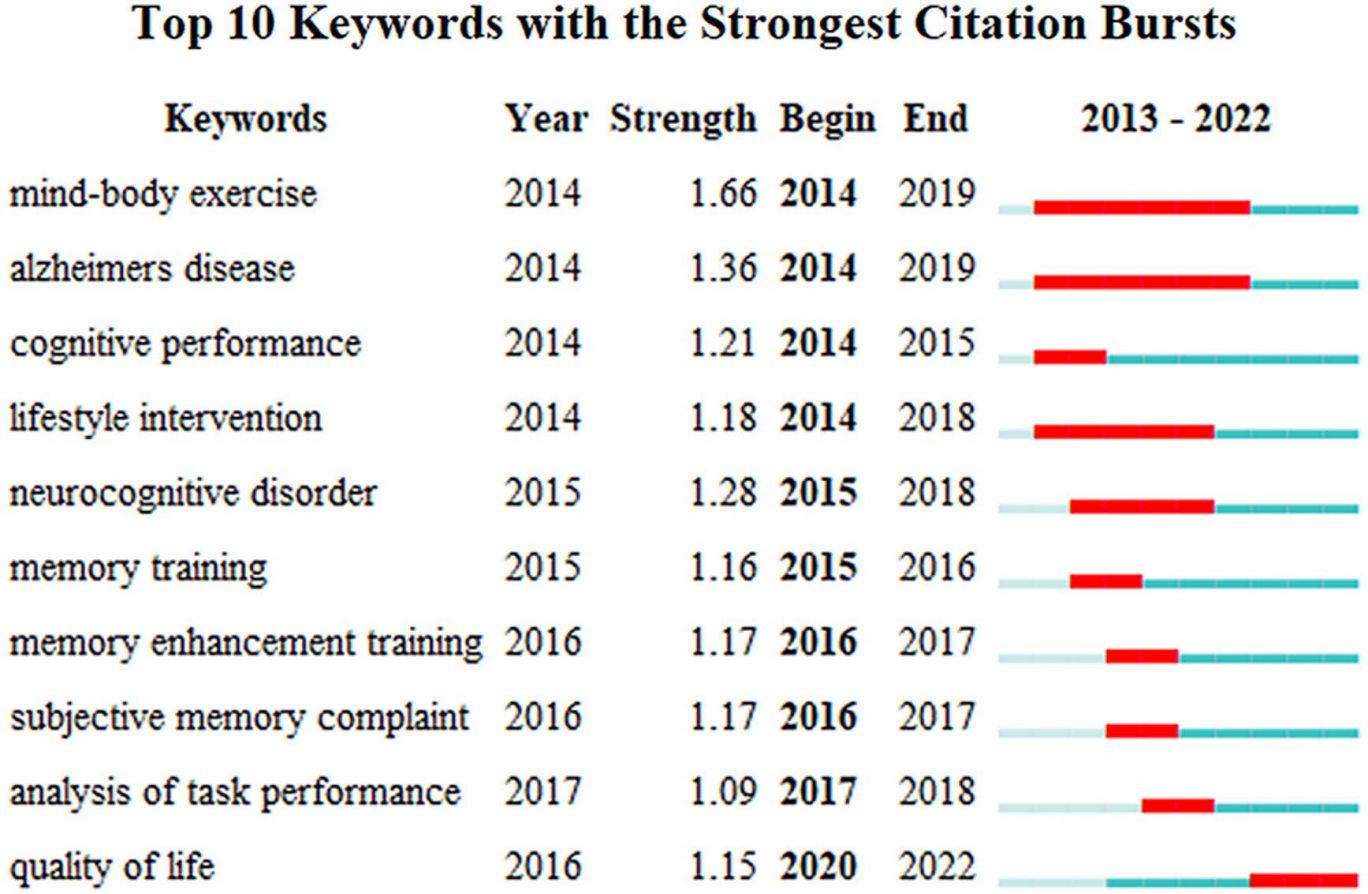
Top 10 keywords with the strongest citation bursts in the field of body–mind exercise for patients with mild cognitive impairment.

The occurrence of the author’s keywords reflects the degree of interest in the field of BME for MCI and can predict future research trends. Meanwhile, the centrality of items was calculated automatically through CiteSpace, while nodes with high centrality represented their critical or turning point significance in a particular research field (43). The 14 most frequently occurring authors’ keywords in the field of BME for MCI are shown in Table 4. MCI was the most frequently occurring keyword and had the highest centrality (0.88). Tai Chi, Older Adults, and Alzheimer’s Disease were also commonly occurring. It is clearly shown in Figure 5 that the top 10 keywords with the strongest citation bursts in the field of BME for MCI in the past decade. The blue line represents the timeline from 2013 to 2022, while the red line represents the duration of the keyword outbreak from 2013 to 2022. In the last decade, body–mind exercise (Burst = 1.66) and Alzheimer’s Disease (Burst = 1.36) were the keywords with the highest citation burst value, as well as the keywords with the longest duration of burst. Since 2020, quality of life has been the keyword with the highest burst value in the field of BME for MCI.

**Table 4:** Top 11 high-frequency keyword in the field of body–mind exercise for patients with mild cognitive impairment.

Rank	Keyword	Quantity	Centrality
1	Mild cognitive impairment	87	0.88
2	Tai Chi	23	0.18
3	Older adults	20	0.07
4	Alzheimer’s disease	15	0.16
=5	Cognitive training	13	0.06
=5	Cognitive impairment	13	0.15
7	Systematic review	12	0.01
=8	Executive function	11	0.03
=8	Cognitive function	11	0.01
=10	Cognitive dysfunction	9	0.09
=10	Physical activity	9	0.04

### 3.6 Reference analysis

The five most cited publications in the field of BME for MCI are shown in Table 5. These five most-cited publications received a total of 719 citations, and only one of the five publications was “Article.” The most cited publication in this field is entitled “Effect of Tai Chi on Cognitive Performance in Older Adults: Systematic Review and Meta-Analysis” (220 times). Wayne et al. (44) systematically reviewed the effectiveness of Tai Chi on overall cognitive function among older adults with normal or pathological cognitive declines (including MCI), and reported that Tai Chi, as a BME, is promising in improving cognitive function in this population. The five most cited articles were all published in journals whose IF was greater than 5, such as COCHRANE DATABASE OF SYSTEMATIC REVIEWS (IF = 11.874), and NEUROSCIENCE AND BIOBEHAVIORAL REVIEWS (IF = 9.062).

**Table 5:** Top 5 most-cited publications in the field of body–mind exercise for patients with mild cognitive impairment.

Rank	Title	First author	Journal	IF	Year	Citation	Type
1	Effect of Tai Chi on Cognitive Performance in Older Adults: Systematic Review and Meta-Analysis	Wayne, P.M.	JOURNAL OF THE AMERICAN GERIATRICS SOCIETY	7.538	2014	220	Review
2	Exercise programs for people with dementia	Forbes, D.	COCHRANE DATABASE OF SYSTEMATIC REVIEWS	11.874	2013	150	Review
3	Physical activity to improve cognition in older adults: can physical activity programs enriched with cognitive challenges enhance the effects? A systematic review and meta-analysis	Gheysen, F.	INTERNATIONAL JOURNAL OF BEHAVIORAL NUTRITION AND PHYSICAL ACTIVITY	8.915	2018	119	Review
4	Maintaining older brain functionality: A targeted review	Ballesteros, S.	NEUROSCIENCE AND BIOBEHAVIORAL REVIEWS	9.052	2015	117	Review
5	Gains in cognition through combined cognitive and physical training: the role of training dosage and severity of neurocognitive disorder	Bamidis, P.D.	FRONTIERS IN AGING NEUROSCIENCE	5.702	2015	113	Article

IF, 2021 Impact Factors.

**Figure 6:**
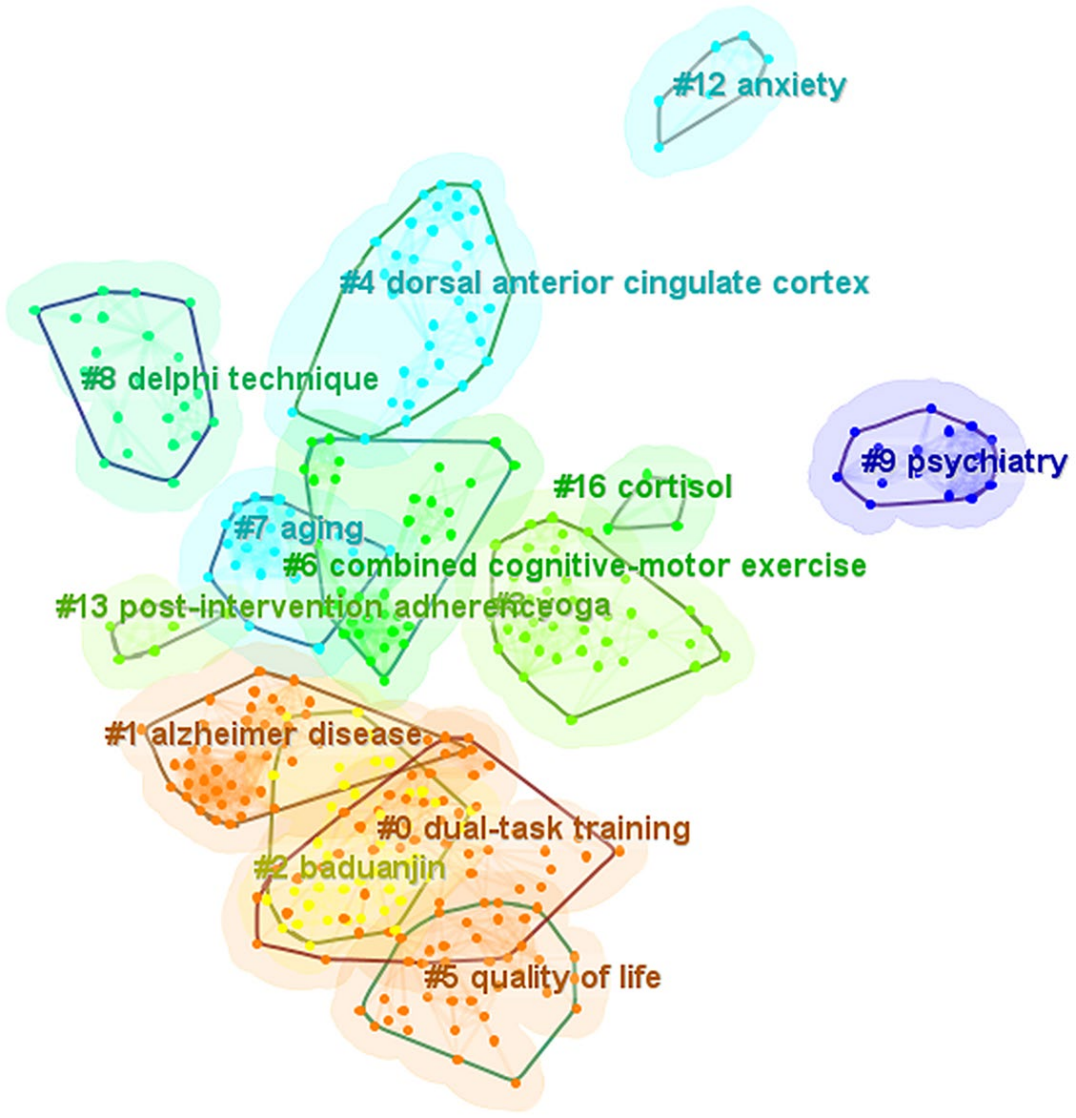
The cluster view of the knowledge map based on reference co-citation analysis in the field of body–mind exercise for patients with mild cognitive impairment.

**Figure 7:**
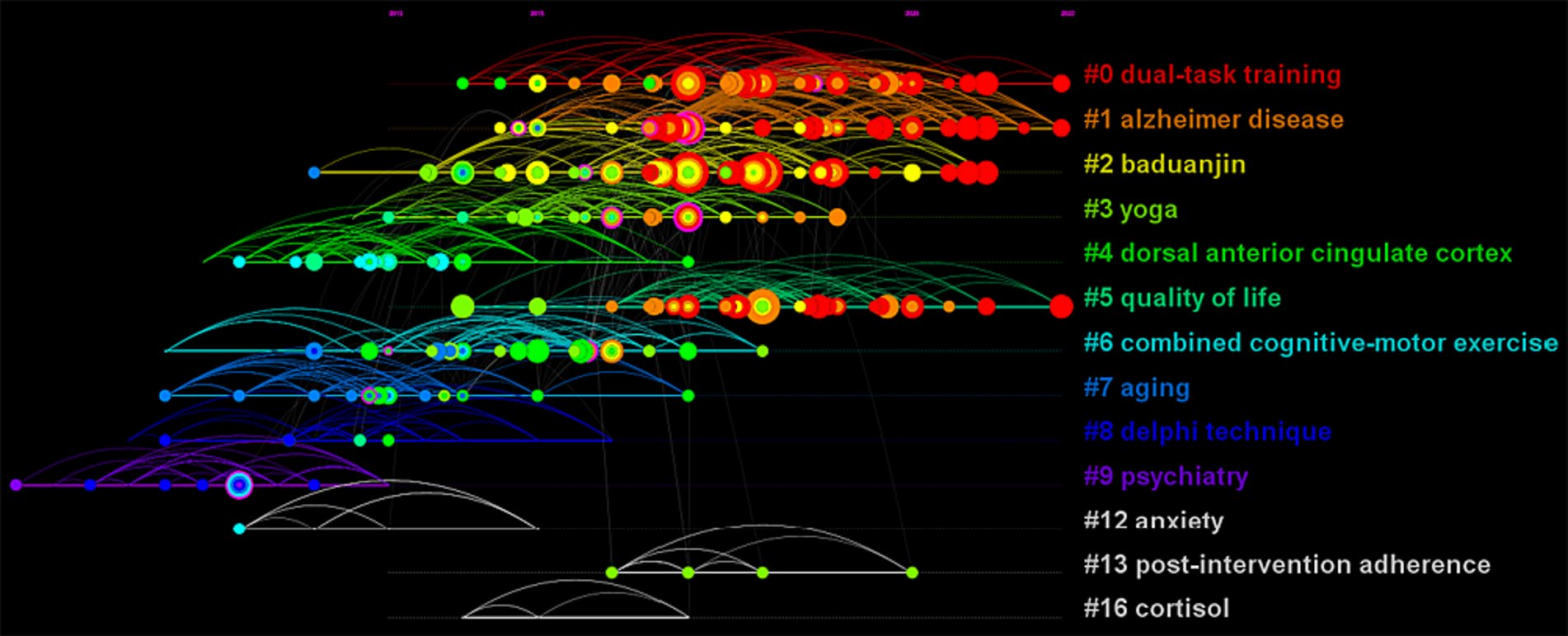
The timeline view of the knowledge map based on reference co-citation analysis in the field of body–mind exercise for patients with mild cognitive impairment.

**Figure 8:**
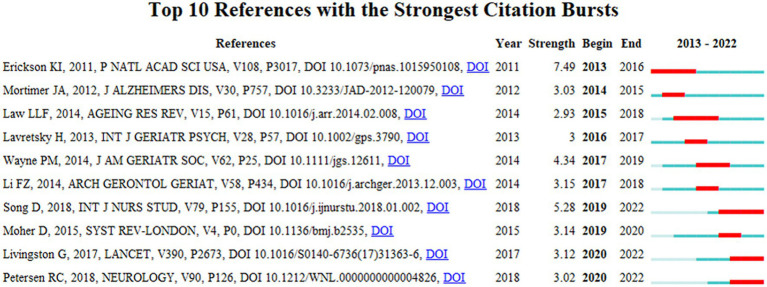
Top 10 references with the strongest citation bursts in the field of body–mind exercise for patients with mild cognitive impairment.

This study conducted an analysis of the reference co-citation network by CiteSpace to explore hotspots and trends in the field of BME for MCI. To measure the validity of clustering strategy, the modularity value (Q) and the weighted average silhouette value (S) were automatically calculated with CiteSpace, while Q > 0.5 and S > 0.7 are the recognized validity thresholds. Therefore, the clustering strategy in this study was valid and reasonable due to its Q = 0.7879 and S = 0.9276. Figure 6 clearly shows the cluster view of the knowledge map of the field of BME for MCI over the past decade, in which 13 clusters were found. Cluster #0 dual-task training had the largest size, followed by #1 Alzheimer’s disease, #2 baduanjin, and #3 yoga. Dual-task training is another description of BME, as we also included this terminology in our search strategy (45). Alzheimer’s disease is the most common type of dementia, and MCI has the potential to progress to Alzheimer’s disease (46). Baduanjin and yoga are two common BME that have been increasingly investigated as potential ways to manage MCI. Meanwhile, a timeline view of clusters, which describes their evolutionary process, is shown in Figure 7. The top 10 co-cited references with the strongest citation bursts are shown in Figure 8, with Erickson et al. (47) having the strongest burst (Burst = 7.49), followed by Song et al. (48) (Burst = 5.28). From 2019 onwards, the publications by Song et al. (48), Livingston et al. (49), and Petersen et al. (50) have been at the frontiers of the burst.

## 4 Discussion

This is the first study to systematically analyze the productivity, research hotspots, and future research trends within the field of BME for MCI over the past decade using a robust bibliometrics approach. The rapid increase in the past decade in both publications and citations in the field of BME for MCI is indicative of the continued steady increase in interest and input from researchers and other stakeholders within the field. Review articles accounted for 40.91% of total publications, which is higher than the results from bibliometric analyses in related fields (33). One possible explanation is that in recent years, having been affected by the COVID-19 pandemic, patients with MCI have been defined by some ethical committees as a vulnerable population and cannot be accessed by intervention studies or epidemiological investigations (51). The proportion of funded research in this area was similar to previous figures for related fields (52), indicating that the field of BME for MCI is preferred by funding agencies and has a large scientific and translational potential. Publications in this field were mainly classified as Geriatrics & Gerontology and Neurosciences, indicating that research in this field was multidisciplinary involving the intersection of geriatrics, neuroscience, and exercise science.

China and the USA were far ahead in terms of national productivity of publications, and Brazil and Thailand also had a high number or quality of publications. Tai Chi, Baduanjin and other BME are popular Chinese traditional exercises, while China also has the largest population of older adults and patients with MCI around the world (53). Therefore, the interest and investment in the field of BME for MCI in China is comprehensible and significant. Nevertheless, developed countries such as Germany and Australia had higher quality research than China. This suggests that developing countries such as China and Brazil need to continue to support research in the field of BME for MCI, and to enhance the quality of research through international exchange and cooperation.

In terms of institutional productivity, although Fujian University of Traditional Chinese Medicine in China was the most productive institution, American institutions occupied eight of the 11 most productive institutions in the field of BME for MCI. Moreover, the level of cooperation between all institutions was low, with some institutions in China having almost no interinstitutional cooperation. Therefore, more international and inter-institutional collaborations are urgently needed for subsequent research in this field. Chen, L was the most productive author in this field and had the strongest collaborative network. Nine of the top 13 most productive authors were from China, but the authors from the USA published higher-quality research. This suggests that Chinese researchers in this field need to improve the quality of their research while maintaining productivity and collaborating across institutions and countries. Furthermore, Frontiers in Aging Neuroscience and JOURNAL OF ALZHEIMER’S are the most popular journals. However, there appear to be some challenges in publishing research in this field in high-quality journals compared to other related fields (33).

Through co-occurrence keyword networks and reference co-citation networks, we identified BME, including Tai Chi and Baduanjin, as hotspots in the field of BME for MCI over the past decade. Tai Chi, a popular traditional Chinese exercise, is a moderate-intensity aerobic exercise that combines both physical and cognitive exercise (54). Research applying Tai Chi, as an intervention for patients with MCI has started relatively early, while Tai Chi has been demonstrated to be effective in enhancing general cognitive performance, memory, attention, and executive function in patients with MCI (55, 56). This may be since it obtains the benefits of both mind and body exercise. First, as moderate-intensity aerobic exercise, tai chi slows age-related brain atrophy, increases cerebral blood circulation, and even alters brain plasticity (57, 58). Second, as a cognitively stimulating activity, Tai Chi requires learning, memorizing, and performing a series of sequential choreographed movements, which promote cognitive functions in participants performing Tai Chi (59). Baduanjin is another traditional Chinese exercise that has been popular in China for more than a thousand years as an important part of the Qigong method (60). Compared to Tai Chi, Baduanjin is a more accessible type of BME and was used later to intervene with patients with MCI but has certainly become a popular intervention in recent years. Previous meta-analyses proposed that Baduanjin also enhanced general cognitive function and executive function among patients with MCI (61, 62). As a BME, Baduanjin may have similar mechanisms of enhancing cognitive function in patients with MCI as Tai Chi. First, Baduanjin improves cardiopulmonary function by controlling breathing, thus enhancing the inhibitory control sublevel of executive function (63). Second, in the practice of Baduanjin, older adults need working memory and executive functions to learn, memorize, and perform motor movements, thus improving their cognitive function (64). Tai Chi and Baduanjin are hot research topics in the field of BME for MCI, they also have greatly promoted the role of traditional Chinese exercise in healthy aging. However, given the difficulty of acquiring these BMEs, there are challenges in promoting and researching them in countries outside of China. This may explain why there is little international or even inter-institutional cooperation in this field.

We identified the future research trends in the field of BME for MCI as evidence synthesis and guidelines through an analysis of bursts in co-occurrence keyword networks and reference co-citation networks. First, over 40% of the publications in this field were review articles. Apart from the limitations of interventions or investigations of patients with MCI during the COVID-19 pandemic, the large number of studies in this field published in Chinese may be another explanation.

However, systematic reviews allow researchers to synthesize evidence from studies published in both English and Chinese. For example, Lin et al. (61) included 13 randomized controlled trials published in Chinese in the meta-analysis. This is an advantage for Chinese researchers but also poses challenges. Mastering both English and Chinese can uniquely position them to synthesize evidence from original research in the field of BME for MCI, but it also limits their ability to internationalize and enhance the quality of evidence synthesis through international collaboration. Furthermore, the large number of publications in this field were published in Chinese and provided only English abstracts limit the development of open science in the field. Another frontier in this field is the guidelines. Livingston et al. (49) in the 2017 report of the Lancet Commission: Dementia prevention, intervention, and care stated that a combination of interventions is needed to delay the progression of MCI to dementia, such as treatment of vascular risk factors, diet, exercise, cognitive and social stimulation, etc. In the updated 2020 report, Livingston et al. (65) indicated that most meta-analyses on the effectiveness of cognitive training for patients with MCI were low-standard, positive, and mostly achieved statistical significance. Currently, there is also evidence that sleep plays an important role in the relationship between exercise and cognitive function (66–68). However, the clinical significance of the results remains uncertain due to the poor standards of the studies and the heterogeneity of the results. Furthermore, the American Academy of Neurology recommended that patients diagnosed with MCI should exercise twice a week, and may have cognitive interventions (50). However, this guideline also notes that the strength of evidence for exercise and cognitive interventions was insufficient and that heterogeneity in outcome measures needed to be reduced in subsequent studies, thus facilitating evidence synthesis and guideline development.

### 4.1 Limitation and further recommendations

Inevitably, there are limitations to this study. First, only one database, WoSCC, was utilized in this study, which may have led to the omission of high-quality studies that exist in other databases but were not indexed by WoSCC. Therefore, software developers are required to upgrade relevant bibliometric tools and algorithms in the future. Second, only English-published studies were included in this study, which may have overlooked high-quality studies published in other languages such as Chinese and Spanish. With advances in bibliometrics techniques as well as international cooperation, it may be possible in the future to minimize linguistic bias. Finally, some high-quality studies may have been overlooked due to late publication, thus not generating hotspots.

## 5 Conclusion

This study performed a bibliometric analysis of BME for MCI in the past decade using a robust methodology, describing knowledge structures, and hotspots, and predicting future research trends in this field. The results of this study help researchers to quickly grasp the knowledge structure in the field of BME for MCI, inform future research, and facilitate inter-institutional and international collaboration. Evidence synthesis and guidelines might be future research trends in this field. Future bibliometric research needs to reduce the limitations imposed by bibliometric techniques, language, and citation delays.

## Data availability statement

The original contributions presented in the study are included in the article/Supplementary material, further inquiries can be directed to the corresponding author.

## Author contributions

JZ: Writing – original draft, Writing – review & editing. ZY: Writing – original draft, Writing – review & editing. HF: Writing – original draft, Writing – review & editing.
