# Supplementary material for: Knowledge structure and future research trends of body–mind exercise for mild cognitive impairment: a bibliometric analysis
Source: Front Neurol. 2024 Jan 23;15:1351741. doi: 10.3389/fneur.2024.1351741 (PMC10844579; doi:10.3389/fneur.2024.1351741)
Supplement: Supplementary file 1 [file Data_Sheet_1.pdf]

Supplementary Material

Knowledge Structure and Future Research Trends of Body-Mind Exercise for Mild Cognitive Impairment: A Bibliometric Analysis

1 Supplementary Data

Search Strategy in the Web of Science Core Collection:

#1 Mild Cognitive Impairment:

(TS=("Mild cognitive impairment" OR "mild cognitive impairments" OR "MCI" OR "cognitive impairment, mild" OR "cognitive impairments, mild")) OR (AK=("Mild cognitive impairment" OR "mild cognitive impairments" OR "MCI" OR "cognitive impairment, mild" OR "cognitive impairments, mild"))

#2 Body-Mind Exercises:

(TS=("Baduanjin" OR "Yijinjing" OR "Yijinjing" OR "Wuqinxi" OR "liuzijue" OR "Tai Chi Quan" OR "Tai Chi" OR "Tai Ji quan" OR "Taijiquan" OR "Tai Ji" OR "Tai-Ji" OR "exergame$" OR "exergaming" OR "cognitive-motor rehabilitation" OR "cognitive-motor exercise" OR "cognitive-motor training" OR "cognitive-motor task" OR "cognitive-motor intervention" OR "cognitive-motor therapy" OR "motor-cognitive rehabilitation" OR "motor-cognitive exercise" OR "motor cognitive training" OR "motor-cognitive task" OR "motor-cognitive intervention" OR "motor-cognitive therapy" OR "dual-task rehabilitation" OR "dual-task exercise" OR "dual-task training" OR "dual-task intervention" OR "dual-task therapy" OR "mind-body rehabilitation" OR "mind-body exercise" OR "mind-body training" OR "mind-body task" OR "mind-body intervention" OR "mind-body therapy" OR "body-mind training" OR "body-mind exercise" OR "body-mind rehabilitation" OR "body-mind intervention" OR "body-mind task" OR "body-mind therapy" OR "virtual reality rehabilitation" OR "virtual reality exercise" OR "virtual reality intervention" OR "virtual reality training" OR "virtual reality task" OR "virtual reality therapy" OR "cognitive behavioral therapy" OR "cognitive behavioral exercise" OR "cognitive behavioral training" OR "cognitive behavioral intervention" OR "cognitive behavioral task" OR "cognitive behavioral rehabilitation" OR "behavioral cognitive therapy" OR "behavioral cognitive exercise" OR "behavioral cognitive

training” OR “behavioral cognitive intervention” OR “behavioral cognitive task” OR “behavioral cognitive rehabilitation” OR “square dance” OR “square dancing” OR “yoga” OR “traditional Chinese exercise” OR “TCE” OR “martial arts” OR “Chinese traditional exercise”))

#1 AND #2
